# Draft Genome Sequence of Bacillus vallismortis Strain BL01, Isolated from *Artemisia lerchiana* Web. Roots

**DOI:** 10.1128/mra.00647-22

**Published:** 2022-10-17

**Authors:** Vladimir K. Chebotar, Maria S. Gancheva, Elena P. Chizhevskaya, Oksana V. Keleinikova, Maria E. Baganova, Alexander N. Zaplatkin, Veronika N. Pishchik

**Affiliations:** a All-Russia Research Institute for Agricultural Microbiology, St. Petersburg, Pushkin, Russia; b Department of Genetics and Biotechnology, Faculty of Biology, Saint Petersburg State University, St. Petersburg, Russia; c Agrophysical Scientific Research Institute, St. Petersburg, Russia; University of Arizona

## Abstract

Some strains of Bacillus vallismortis have been reported to be efficient plant-growth-promoting bacteria as well as inducers of systemic resistance. Here, we report the draft genome sequence of Bacillus vallismortis strain BL01, isolated from the roots of Artemisia lerchiana Web.

## ANNOUNCEMENT

Bacillus vallismortis can produce metabolites with strong growth inhibition activity against phytopathogenic fungi (1–3) and bacteria (4) and can increase plant protection against viruses (5). Also, Bacillus vallismortis is able to produce growth-regulating substances that increase yields (3, 5, 6). B. vallismortis strain BL01 was isolated from the roots of Artemisia lerchiana Web. from the Astrakhan region, Russia (46.519511°N, 47.915334°E). Plant roots were disinfected with tap water for 30 s followed by 70% ethanol for 5 min and then 15% H_2_O_2_ for 10 min and sterile water for 2 min 5 times and subsequently crushed with a mortar and pestle under sterile conditions. Aliquots of 100 mL of the resulting plant juices were plated onto a 1/20 dilution of tryptic soy agar (TSA; Difco Laboratories, MI, USA) plates. The sterility check consisted of aliquots of water from the last rinsing that were plated onto 1/20 TSA according to methods reported previously (7). Plates were incubated at 28°C for 3 days. Strain BL01 was grown in LB medium at 30°C overnight. The total cellular DNA was isolated from a single colony using the cetyltrimethylammonium bromide (CTAB)-NaCl method (8). Paired-end reads were generated using the Nextera DNA Flex kit (Illumina, USA). The complete genome was sequenced using the Illumina HiSeq 2500 technology. A total of 415,271 reads were generated, with an average length of 280 bp. FastQC v0.11.9 (9) was used to assess the quality of the reads. Trimmomatic v0.39 (10) was used for trimming low-quality sequences. The trimmed reads were classified using Kaiju v1.8.2 against the RefSeq genomes under maximum exact match (MEM) mode to maximize exact matches (11). The reads were *de novo* assembled using SPAdes v3.14.1 (12) with the “–careful” option. The nucleotide compositions of contigs and scaffolds were determined using Seqtk v1.3-r106 (https://github.com/lh3/seqtk). The contigs were mapped to the Bacillus vallismortis strain Bac111 (GenBank accession number CP033052.1) genome by CONTIGuator v2.7 (13). Default parameters were used for all software unless otherwise specified. Contigs were assembled into a single scaffold using Ragtag v2.1.0 (gaps filled with N’s) (14). Evaluation of the genome assembly was performed with Quast v5.1.0 (15) and BUSCO v5.2.2 (bacillales_odb10) (16). The final assembly contained 4,115,091 bp in 1 scaffold, and the average GC content of the assembly was 44.01%. The BUSCO results recovered 99.8% of the *Bacillales* database. Genome annotation was performed using the NCBI Prokaryotic Genome Annotation Pipeline (PGAP-6.1) (17). A total of 4,029 protein-coding sequences (CDSs) and 80 tRNA genes were predicted. The complete genome sequence of Bacillus vallismortis BL01 will contribute to revealing the role of Bacillus vallismortis in plant growth and physiology.

### Data availability.

All data are available in the National Center for Biotechnology Information database under BioProject accession number PRJNA809498. The raw reads were deposited in the Sequence Read Archive under accession number SRR18107534. The GenBank nucleotide sequence accession number is CP092751.
